# A New Biological Feature of Natural Killer Cells: The Recognition of Solid Tumor-Derived Cancer Stem Cells

**DOI:** 10.3389/fimmu.2016.00179

**Published:** 2016-05-10

**Authors:** Rossana Tallerico, Cinzia Garofalo, Ennio Carbone

**Affiliations:** ^1^Tumor Immunology and Immunopathology Laboratory, Department of Experimental and Clinical Medicine, University “Magna Graecia” of Catanzaro, Catanzaro, Italy; ^2^Department of Microbiology, Cell and Tumorbiology (MTC), Karolinska Institutet, Stockholm, Sweden

**Keywords:** NK cells, CSCs, MHC class I, immunotherapy, solid tumor

## Abstract

Natural killer (NK) cells are classified as a member of the innate lymphoid cells (ILCs) group 1. ILCs have been recently identified and grouped on the basis of their phenotypical and functional characteristics. They are effectors of innate immunity and are involved in secondary lymphoid organ generation and tissue remodeling. NK cells are powerful cytotoxic lymphocytes able to recognize and eliminate tumor- and virus-infected cells by limiting their spread and tissue damage. The recognition of tumor cells is mediated by both activating and inhibitory receptors. While in hematological malignancies the role played by NK cells is widely known, their role in recognizing solid tumors remains unclear. Recently, tumor cell populations have been divided into two compartments: cancer-initiating cells (CICs) or cancer stem cells (CSCs) and senescent tumor cells. Here, CSC will be used. CSCs are a small subset of malignant cells with stem-like properties that are involved in tumor maintenance and recurrence due to their ability to survive to traditional therapies; they are, moreover, poorly recognized by T lymphocytes. Recent data showed that NK cells recognize *in vitro* cancer-initiating cells derived from colon cancer, glioblastoma, and melanoma. However, more *in vivo* studies are urgently required to fully understand whether these new antitumor NK cells with cytotoxic capability may be considered in the design of new immunotherapeutic interventions.

## NK Cells

The large granular innate lymphoid cells (ILCs) are named as natural killer (NK) cells for their protective and cytotoxic role in infectious and tumor diseases. They play an active role in transplant rejection and in pathological conditions. The main effectors mechanisms of NK cells are lymphocytotoxicity and proinflammatory cytokine production. Recently, they were classified in Group 1 of the ILCs (1).

In human, NK cells are phenotypically characterized by the surface expression of molecules like CD56 and CD16 (FcγRIII) and by the absence of CD3, while in mice, they are defined as cells expressing NK1.1, DX5 and not expressing CD3. NK cells rely on a set of germ-line encoded activating and inhibitory receptors. The activating receptors include CD16, NKG2D, DNAM-1, and NKp46, all common to both human and mouse, while the other NK receptors (NCRs: NKp44 and NKp30) are expressed solely by human NK cells. Apart from the CD16 receptor recognizing Fc domain antibodies, the other receptors interact with ligands on infected and transformed cells. Molecules associated with a “stress signature,” such as, MICA, MICB, ULBPs, and Hsp90 in human and Rae-1, MULT-1, and H60 in mouse, have been identified as major activating ligands (2). Other activating NK ligands are PVR and NECTIN-2 bound by DNAM-1, expressed both in human and mice. NCR ligands are still unknown, except for NKp30, whose ligands are B7-H6, BAG-6, and Gal-3 (3, 4).

The inhibitory receptors of NK cells interact with major histocompatibility complex (MHC-I) molecules. In human, most of these receptors belong to the family of killer cell immunoglobulin like receptors (KIRs) that directly bind to certain HLA-A, -B, and -C alleles. KIRs are highly polymorphic receptors that interact with MHC-I molecules and elicit inhibitory or activating signals. Their name is correlated with the number of Ig-like domains in their extracellular regions and with the presence of a long (inhibitory) or short (activating) cytoplasmic tail. Other types of inhibitory and activating NK cell receptors are the lectin-like heterodimers of NKG2 family. The best-known members are the inhibitory heterodimer CD94/NKG2A and its activating counterpart CD94/NKG2C, both recognizing the non-classical MHC class I molecule, HLA-E. In mice, the inhibitory receptors belong to the superfamily of lectin-like type II integral membrane proteins, known as Ly49 and CD94/NKG2A receptors.

The lack or reduced expression of MHC-I on the target cells’ surface leads to “missing self” recognition. There are clinical observations and ongoing clinical trials based on the idea that NK cells transferred from one individual to a non-MHC-I-matched patient can exert antileukemic effects based on “missing self” recognition (5).

In both species, the fully matured highly cytotoxic NK phenotype is acquired after a, still poorly understood, MHC class I molecule-dependent ontogenic process. The NK cells ontogeny process has been termed “education – licensing – arming/disarming” (6, 7). Recently, Kärre and coworkers have proposed to explain the NK cells’ education in the “Rheostat Model” (8). The “Rheostat Model” postulates that education of NK cells represents a tuning mechanism that goes on continuously and reversibly accordingly to the MHC environment, even after NK cell maturation. This model has implications for immunotherapy based on exploiting “missing self” reactivity when NK cells are transferred to a cancer patient from a healthy, non-MHC-matched individual. If the NK cells are “re-tuned” in the new environment, as proposed in the rheostat model, this may affect their efficacy to react against the recipient’s leukemia cells and can influence the reactivity of NK cells against tumor cells in different situations. It has been shown that NK cells can exert antitumor effects in the new host environment. Recently, Wagner and coworkers provided further support to the rheostat model demonstrating that NK cells infused in a recipient become tolerant to healthy cells representing “missing self” in the new environment, while the re-tuned NK cells will retain activity against tumor cells representing “missing self,” due to a higher expression of ligands for activating NK receptors on tumor cells (9).

Another new concept that has contributed to change our knowledge about the NK cells biology derives from the observations made during human and mice CMV infection, where NK cell subsets recognizing CMV-infected cells expanded. The expanded CMV-specific population has been proposed to be a relevant NK cell memory subset able to provide immunity in the second infection round (10–12).

Therefore, the information that are now available on the NK cells biology are pushing the field toward their use in clinical settings.

## NK and Cancer

The history of NK cells has evolved closely related with the history of cancer immunotherapy since they were discovered in the 1970s for their capability to target and kill cancer cells. NK cells preventing leukemia relapse in mismatched hematopoietic stem cell transplant (HSCT) patients stirred a renewed interest in the use of NK cells in the fight against cancer. They are the most efficient effectors against tumors and are considered suitable candidates for adoptive immunotherapy of both hematological and non-hematological malignancies (13–15).

Nearly 50 years after their discovery, we are now starting to realize the potential of these NK cells in tumor immune therapy trials.

## Cancer Stem Cells

The processes underlying the etiology of cancer have been the source of several theories for a century (16, 17).

It was demonstrated that any cell exposed to genotoxic stress acquires multiple genetic mutations and may give rise to a tumor (18). More recently, Tomasetti and Vogelstein demonstrated that stochastic influences are the major contributors to cancer overall, often even more than hereditary or external environmental factors. Many genomic changes occur simply by chance during DNA replication rather than as a result of carcinogenic factors. Considering the number of mutations needed for a malignant transformation, it is conceivable that these can only accumulate in long-lived cells (stem cells) (19).

Therefore, cancer stem cell hypothesis postulates that only a small subset of cells, termed cancer-initiating cells (CICs) or cancer stem cells (CSCs), is able to give rise to and maintain tumors (20–22), even though for transplanted murine tumors, such as melanoma, the relevance of CSCs has been questioned (23).

Cancer stem cells constitute a self-renewing stem cell-like population, having an asymmetric proliferating pattern responsible for the progression, metastasis, resistance to treatment, and recurrence of several tumors (24–26).

Compelling data support the cancer stem cell model in various human cancers including malignant embryonic tissues cell cancers (27, 28), leukemias (29, 30), breast cancers (31), brain cancers (32), and colon cancers (33–35). Based on the different tumor types, the CSCs were identified by the expression of different markers, such as CD133^+^ CD166^+^, CD44^+^, CD29^+^, CD24^+^, EpCAM, Lgr5, aldehyde dehydrogenase, and a few others (36).

The main feature of the CSCs is that a small amount of them (up to 100 cells) may transfer disease upon transplantation into immune-compromised NOD/SCID mice (34, 35). Some markers have been identified that distinguish the leukemogenic/tumorigenic cancer cells (leukemogenic CSC) from the bulk populations of non-leukemogenic/tumorigenic cells (non-leukemogenic CSC). The ability to predict which cells are tumorigenic based on marker expression indicates that the tumorigenic cells are intrinsically different from non-tumorigenic cancer cells.

## NK and CSC

Cancer stem cells have been suggested to be responsible for the hematological metastatic spreading of solid tumors (37). The asymmetrical growth and their low rate of proliferation make them resistant to conventional radiotherapy and chemotherapy. Therefore, to prevent CSCs contribution in sustaining tumor progression, several groups have evaluated whether the effector immune cytotoxic cells (NK cells, CD8 T cells, and γδ T cells) could eliminate the tumor stem compartment. However, the low level of MHC class I expression reported for CSCs predicts a low efficiency of CSCs targeting by CD8^+^ cytotoxic T lymphocytes, while recent data demonstrate that once the CSC is sensitized with bisphosphonate zoledronate, the human γδ T cells could efficiently target CSCs *in vitro* (38). The cytotoxic interaction was regulated *via* TCR and to a lesser extent by NKG2D receptors (38). On the other hand, data have shown that NK cells, mainly *in vitro*, selectively kill human colon-derived CSCs without any pharmacological pretreatment (39), melanoma (40), and glioblastoma (41). The molecular mechanisms leading the NK cell recognition of CSC have been partially elucidated.

In our laboratory, we demonstrate that freshly purified autologous and allogeneic NK cells show a robust cytotoxic effect on CIC derived from colorectal carcinoma cells (CRC), whereas the CRCs were less susceptible to the NK lysis, Tallerico et al. (39). The capability to preferentially recognize and kill colorectal-CIC is correlated with a higher expression of NCR ligands (in particular, NKp30 and NKp44) and a lower expression of MHC class I on the surface of CSCs compared to their tumor counterparts (39).

In another pathological setting, Pietra and colleagues (40) analyzed different melanoma cells derived from metastatic primary cultures or from established cell lines. These cells have a highly heterogeneous expression of different stem cell-associated markers including CD133, c-kit/CD117, and p75 neurotrophin receptor/CD271. IL-2-activated NK cells are able to recognize and lyse melanoma cells enriched CSC through the involvement of a different combination of activating NK receptors. Indeed, NK cells efficiently kill melanoma cells resistant to radiotherapy, either with ability to form melanospheres *in vitro* or having the stem cell-associated marker CD133 expression, underlying the possibility to use a novel NK-based immunotherapeutic strategy to eliminate CSC (42).

This was demonstrated also by Castriconi and colleagues (41) in glioblastoma patients, in which CSCs were isolated and characterized for typical markers of neural stem cells. They were capable of partial multilineage differentiation *in vitro* and gave origin to infiltrating tumors when orthotopically injected in NOD/SCID mice. These cells, characterized by stem cell-like properties, are well killed by allogeneic and autologous NK cells activated by IL-2 or IL-15. The NK-mediated killing of glioblastoma cells (GBM) has been identified by the low levels of HLA class I both classical (HLA-A, -B, -C) and non-classical (HLA-E) molecules and by the expression of DNAM-1 and NKp46 activating NK receptors. Moreover, most of GBM cells express different amounts of NKG2D ligands, while all GBM express PVR and NECTIN-2 (DNAM-1 ligands) (41).

The Pietra and the Castriconi studies provide novel therapeutic approaches based on the use of activated NK cells useful to eradicate tumor cells residuals after surgery or a traditional therapy.

Thus, these combined studies confirm that the molecular mechanisms behind the NK cell-mediated recognition of CSC rely on their loss or low MHC class I expression and increased amounts of activating NK ligands, on their cell surfaces. It should be noted that the low expression of MHC class I is not always the main mechanism by which NK cells recognize tumor cells. Tumors could lose or not their MHC class I expression, however, they could acquire NK receptor activating ligands on the cell membrane leading to a specific NK cell recognition.

A crucial issue to exploit the NK cells to target CSC is to validate these *in vitro* observations in experimental *in vivo* models.

So far, very few *in vivo* studies address the potential of NK cells to ablate the CSC compartment from the tumor population.

Recently, Ames and colleagues (42) showed that NK cells kill CSCs from different kinds of tumors, through the interaction of the NKG2D activating receptor with its ligand (MICA/B). They have shown, *ex vivo* and *in vivo*, the efficacy of the NK cell therapy to target CSCs. They implanted human tumor cell lines in NOD SCID gamma (NSG) mice and then injected activated NK cells. After NK infusion, they sacrificed mice and observed the number of tumor colonies when homogenized lung cells were plated in a lung colony formation assay. The colonies that did develop in NK cell-treated mice showed an impressive reduction in size, consistent with NK cell targeting of stem-like tumor cells. In mice with established orthotopic pancreatic tumors that were treated with NK infusion, Ames observed by bioluminescence, the reduction of the tumor size, demonstrating the therapeutic efficacy of NK cells (42). All the discussed data are summarized in Table 1.

**Table 1:** **Phenotypes and NK cells recognition pattern of cancer stem cells and related tumors**.

Human tumor	Type	PVR	NECTIN-2	MICA	MICB	ULBPs	NCR ligands	HLA	NK cell recognition
Colorectal carcinoma (39)	Tumor	NC	NC	NC	NC	NC	+/−	+	+/−
							NKp30L	HLA-A, -B, -C	
							NKp44L		
	Cancer stem cells	NC	NC	NC	NC	NC	+	+/−	++
							NKp30L	HLA-A, -B, -C	
							NKp44L		
Melanoma (40)	Tumor	+/−	+/−	+/−	ND	+/−	ND	+	+
						ULBP2		HLA-A, -B, -C	
	Cancer stem cells	+/−	+/−	+/−	ND	+/−	ND	−	+
						ULBP3		HLA-A, -B, -C	
Glioblastoma (41)	Tumor	−	−	−	−	−	ND	+	+/−
								HLA-E	
	Cancer stem cells	+	+	+/−	+/−	+/−	ND	+	++
								HLA-A, -B, -C	
								HLA-E	
Pancreatic adenocarcinoma (42)	Tumor	ND	ND	−	−	ND	−	NC	+/−
	Cancer stem cells	ND	ND	+	+	ND	−	NC	++
Sarcoma Ewing (42)	Tumor	ND	ND	−	−	ND	−	NC	+/−
	Cancer stem cells	ND	ND	+	+	ND	−	NC	++
Liposarcoma (42)	Tumor	ND	ND	ND	ND	ND	ND	ND	+/−
	Cancer stem cells	ND	ND	ND	ND	ND	ND	ND	++
Breast carcinoma (42)	Tumor	ND	ND	ND	ND	ND	ND	ND	+/−
	Cancer stem cells	ND	ND	ND	ND	ND	ND	ND	++
Glioblastoma (42)	Tumor	ND	ND	ND	ND	ND	ND	ND	+/−
	Cancer stem cells	ND	ND	ND	ND	ND	ND	ND	++

*NC, no change between tumor and CSC; ND, not detected*.

## Conclusion

Different promising strategies to directly attack CSCs have been proposed using antibody-based therapies (43) by blocking pathways important for homing and engraftment (CD44), self-renewal (CD27), protection against phagocytosis (CD47), or by strongly activating CD8^+^ T cells (CTLs) specific for CSC antigens or simply by forcing CSC into cell cycle. However, their therapeutic success has been limited. This calls for new approaches to target the CSCs compartment as a valid anticancer therapy.

The recent findings discussed here showed that the immune system, in particular the innate part (dendritic, NK, and γδ T cells), can efficiently cooperate to eliminate CSC; this allow us to hypothesize that a promising avenue to eradicate CSC might lie in harnessing NK and γδ T cells. However, the *in vivo* functional outcome of NK–stem cell interplay may also result in NK anergy (44, 45). Such NK cell dysfunctional plasticity is believed to have a major impact in NK cell-based immunotherapeutic approaches and deserves a deeper understanding through *in vivo* models.

Several studies (46) have demonstrated that chemotherapy either induce or increase the CSCs susceptibility to NK- and γδ T cell-mediated killing. Therefore, combination of immune-based therapies with chemotherapy could be beneficial in the treatment of many cancers.

The current failure of standard therapies is attributed to a small fraction of the primary cell population with stem-like characteristics (CSC), such as self-renewal and differentiation. So, it is imperative to target all CSCs within the tumor to prevent relapse. Even though different aspects of CSCs have been explored in recent targeting strategies, their success has been very limited probably because an exhaustive comprehension of their basic biology and evolution is far from being clarified.

In order to eradicate the CSCs, we need to act at multiple levels: increasing their sensitivity to chemotherapy and to novel compounds, stimulating or reactivating a tumor-specific immune response selectively directed against the CSCs, enhancing the efficacy of therapies currently in use with a selective induction of the immune response, and testing the antitumor and immune-stimulating properties of new compounds. Further investigations are necessary to better understand the basic biology of immune recognition of CSC, which may be rapidly translated into innovative therapeutic approaches for the treatment of different forms of cancer.

Moreover, in accordance with our and Pietra, Castriconi, and Ames studies, NK-mediated killing is a possible candidate for targeting CSCs following the depletion of non-CSCs by anti-proliferative therapies. These studies focused on possible strategies to eradicate CSCs from established tumors; taking together the data produced, it is conceivable to think that NK cells can selectively recognize CSC compartments of several solid tumors, *in vitro*. At this stage, however, only a few studies are available on the *in vivo* NK cell-mediated CSC eradication and tumor control.

Therefore, more *in vivo* studies are necessary to address the hypothesis that NK cells can eradicate the CSCs population in a pathophysiological setting and to establish new immune-intervention strategies in solid tumors that can be readily transferred in new clinical trials.

## Author Contributions

All the authors contributed in the writing of this review.

## Conflict of Interest Statement

The authors declare that the research was conducted in the absence of any commercial or financial relationships that could be construed as a potential conflict of interest.
